# Role of echogenic foci in ultrasonographic risk stratification of thyroid nodules: Echogenic focus scoring in the American College of Radiology Thyroid Imaging Reporting and Data System

**DOI:** 10.3389/fonc.2022.929500

**Published:** 2022-08-29

**Authors:** Renxu Li, Zhenwei Liang, Xiangyu Wang, Luzeng Chen

**Affiliations:** Department of Ultrasound, Peking University First Hospital, Beijing, China

**Keywords:** echogenic foci, ultrasonography, ACR TI-RADS, thyroid nodules, malignancy

## Abstract

**Background:**

Although echogenic foci may raise malignancy rates in thyroid nodules, the association between peripheral calcification or macrocalcification and thyroid carcinoma is controversial. We evaluated the malignancy probability of various echogenic foci and explored whether the method of determining a thyroid nodule’s point score in the echogenic focus category of the American College of Radiology (ACR) Thyroid Imaging Reporting and Data System (TI-RADS) is reasonable.

**Methods:**

We retrospectively evaluated 819 patients with 852 nodules. The patterns of echogenic foci on ultrasonography were classified into the following four categories: punctate echogenic foci, macrocalcification, peripheral calcification, and multiple different types of echogenic foci. The core needle biopsy results were divided into two groups: benign and malignant or suspicious for malignancy.

**Results:**

Among the 852 nodules, 471 (55.3%) had echogenic foci on ultrasonography. Of these nodules, there was no significant statistical difference in the malignant or suspicious for malignancy rate between nodules with peripheral calcification and those with macrocalcification [40.0% (8/20) *vs*. 30.6% (11/36), respectively; *p* = 0.474]. The incidence of malignancy or suspicious for malignancy for nodules with peripheral calcification, macrocalcification, or multiple different types of echogenic foci was significantly lower than the incidence for punctate echogenic foci alone, with odds ratios of 0.265 [95% confidence interval (CI): 0.105–0.667; *p* = 0.005], 0.175 (95% CI: 0.083–0.368; *p* = 0.000), and 0.256 (95% CI: 0.136–0.482; *p* = 0.000), respectively.

**Conclusion:**

We found no significant statistical difference in the risk of malignancy or suspicious for malignancy rate between peripheral calcification and macrocalcification in thyroid nodules. We observed that nodules with multiple different types of echogenic foci were not associated with higher malignant or suspicious for malignancy rates compared with nodules with punctate echogenic foci alone.

## Introduction

Thyroid nodules are detected commonly in adults by neck ultrasonography (US), and approximately 5%–15% of these nodules are malignant (1, 2). US has been used to compare the presence and patterns of echogenic foci in thyroid nodules. Although thyroid echogenic foci are associated with benign and malignant thyroid tumors, echogenic foci may raise suspicion for thyroid cancer (3); specifically, 62.5%–81.8% of nodules with microcalcification were eventually identified as malignant (4, 5). Previous studies showed that the malignancy rate of thyroid nodules with coarse calcification was 3%–40% (6, 7). Although studies have suggested that peripheral calcification is associated with malignancy, the correlation with an increased likelihood of malignancy varied (8, 9).

In the American Thyroid Association management guidelines for adult patients with thyroid nodules and differentiated thyroid cancer, microcalcification is one of the US features associated with a high suspicion of malignancy (10). Similarly, punctate echogenic foci (suspected microcalcification) are also defined as a positive feature in the 2020 Chinese guidelines for US malignancy risk stratification of thyroid nodules (11). Compared with the American Thyroid Association management guidelines and the Chinese guidelines, the American College of Radiology (ACR) Thyroid Imaging Reporting and Data System (TI-RADS) (12) classifies echogenic foci into three categories: punctate echogenic foci (may have small comet-tail artifact), peripheral calcifications, and macrocalcifications, which are assigned 3 points, 2 points, and 1 point, respectively. All classifications that apply to the echogenic foci are chosen when determining the TI-RADS level. In our study, we aimed to evaluate the malignancy probability of punctate echogenic foci, peripheral calcifications, macrocalcifications, and nodules with multiple different types of echogenic foci. We also evaluated whether the method of determining a nodule’s score for the category of echogenic foci in the ACR TI-RADS is reasonable.

## Materials and methods

### Subjects

**Figure 1:**
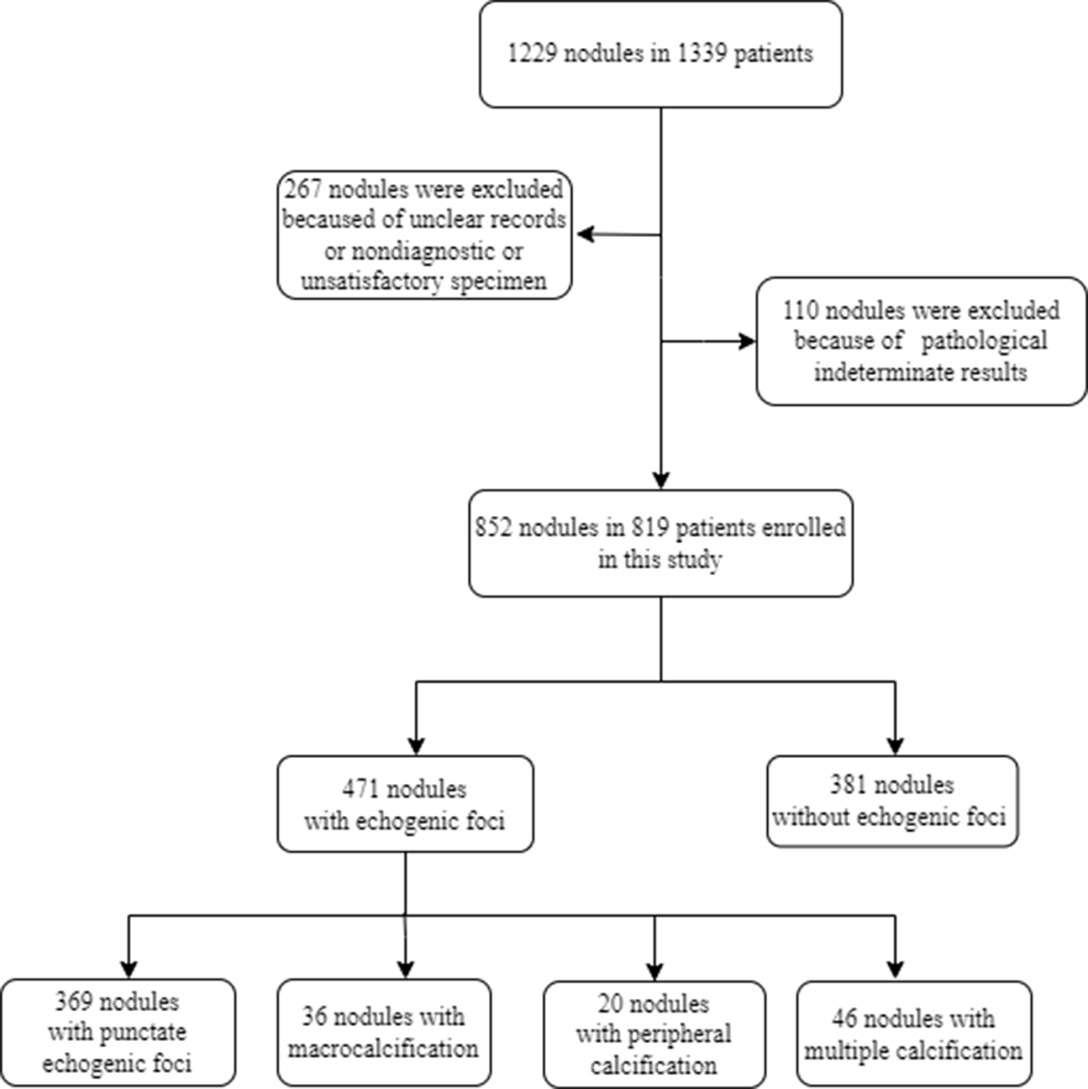
Flowchart of this paper.

We retrospectively collected data for patients undergoing thyroid US and US-guided core needle biopsy (CNB) from March 2013 to March 2015 (**Figure 1**). The inclusion criteria were as follows: 1) patients whose thyroid nodules have more than one of the following US features: hypoechoic or very hypoechoic, anteroposterior dimension/transverse dimension ratio (AP/T) >1, echogenic foci, irregular margin, or ill-defined margin; 2) maximal nodule dimension ≥5 mm; 3) patients whose nodule biopsy was performed in our hospital; 4) if other US features could not be determined due to dense calcification, CNB was performed when the nodule was greater than 5 mm. The exclusion criteria were as follows: 1) thyroid nodules with unclear records in the radiology database; 2) nondiagnostic or unsatisfactory specimen was acquired by CNB; 3) nodules with CNB pathological indeterminate results. All patients were informed of the possible risks during CNB and provided a signed informed consent. This study protocol was approved by the ethics committee of our hospital.

### Ultrasonographic examination

**Figure 2:**
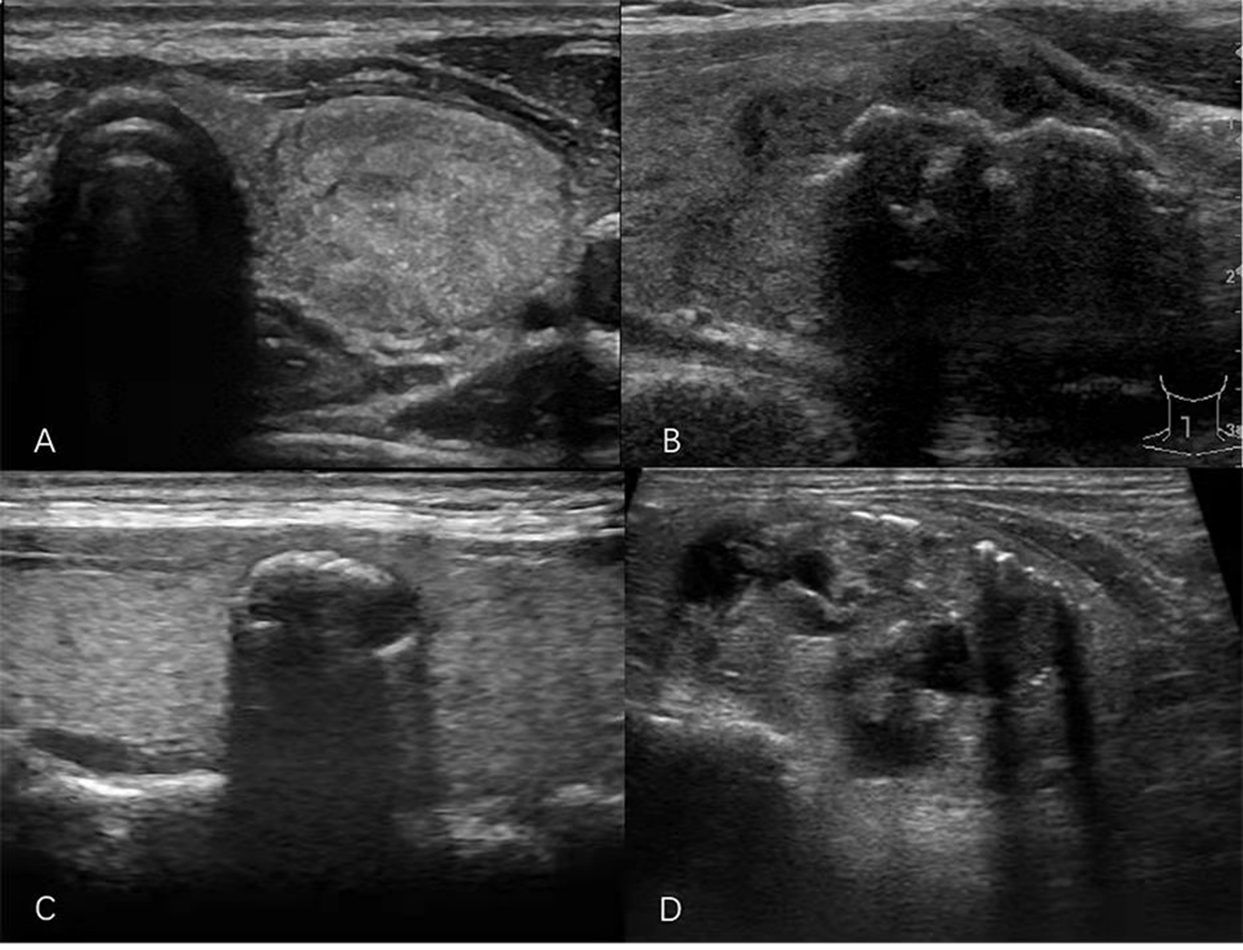
Sonograms of thyroid nodules showing different patterns of echogenic foci. **(A)** Punctate echogenic foci. **(B)** Macrocalcification. **(C)** Peripheral calcification. **(D)** Multiple different calcification types.

US examination of the thyroid gland was performed with the iU22 US system (Philips, Amsterdam, Holland), Logiq E9 system (General Electric, Boston, MA, USA), Siemens S2000 system (Siemens, Erlangen, Germany), ALOKA α7 system (Hitachi Aloka Medical, Tokyo, Japan), or Mylab 90 system (Esaote Medical System, Genova, Italy), equipped with a high-frequency wide-band linear array probe with a frequency range of 7–12 MHz. Nodule location, diameter, shape, echogenicity, composition, margin, and echogenic foci were examined. The nodule shape was categorized as AP/T >1 (a ratio of >1 in the anteroposterior dimension to the transverse dimension in the transverse plane) or AP/T < 1. The level of echogenicity of the solid components of nodules was classified as follows: hyperechoic (increased echogenicity relative to thyroid parenchyma), isoechoic (similar echogenicity relative to thyroid parenchyma), hypoechoic (decreased echogenicity relative to thyroid parenchyma), or very hypoechoic (decreased echogenicity relative to the strap muscles of the neck). Composition describes the internal components of a nodule and was categorized as solid (pure solid or nearly entirely solid), cystic (no obvious solid content), mixed cystic and solid, or spongiform (multiple tiny cystic spaces occupy the entire nodules without aggregated solid tissues). Margins were classified as smooth, irregular, ill-defined, or extrathyroidal extension (nodule extends through the thyroid capsule). The patterns of the echogenic foci were classified into the following four categories: punctate echogenic foci (≤1 mm) with or without posterior shadowing (according to ACR TI-RADS, punctate echogenic foci may have some small comet-tail artifacts located in the solid components); macrocalcification (>1 mm), usually accompanied by posterior shadowing; peripheral calcification (located at the periphery of the nodules and that might appear as a continuous or discontinuous ring or arc involving more than one-third of the margin); and multiple different types of echogenic foci (present as two or more types of echogenic foci) (**Figure 2**). All sonographic images (static and video clips) were retrospectively interpreted jointly by two radiologists with more than 5 years and more than 10 years of experience. If the radiologists’ opinions regarding the findings of echogenic foci or other US features included in the ACR TI-RADS were not in consensus, the images were interpreted by a third radiologist who has performed thyroid US for more than 20 years.

### Core needle biopsy pathology

Thyroid nodule CNB was performed by the same experienced radiologist team with an 18-gauge needle (Bard, Tempe, AZ, USA) under US guidance. One or two specimens were obtained from each nodule. The CNB interpretation was according to the Korean CNB pathology reporting system (13), as follows: benign lesion (II), indeterminate lesion (III), follicular neoplasm or suspicious for a follicular neoplasm (IV), suspicious for malignancy (V), or malignant (VI). Combining classifications III and IV as the indeterminate category, which was excluded from our analysis, and classifications V and VI as the suspicious for malignancy or malignant category, we evaluated the differences in US features, namely, echogenic foci, between the benign category and the suspicious for malignancy or malignant category.

### Statistical analysis

Data were analyzed using SPSS software (IBM Corp., Armonk, NY, USA). Descriptive statistics were expressed as mean ± standard deviation or median (interquartile range) for continuous variables, depending on the distribution type, and as the number of cases and percentages for nominal variables. Data for echogenic focus type were compared using the chi-square or Fisher’s exact test. Regression analysis was used to assess the diagnostic capability of the different types of echogenic foci for predicting malignancy with reference to CNB pathology. The data were presented as odds ratios (ORs) and 95% confidence intervals (CIs). A *p* value <0.05 was considered statistically significant.

## Results

A total of 1,229 consecutive nodules in 1,139 patients were biopsied. Among these, 267 thyroid nodules with unclear records in the radiology database or nondiagnostic or unsatisfactory specimen acquired by CNB and 110 nodules with CNB pathological indeterminate results were excluded from this study. Finally, 819 patients with 852 nodules were included in the study, with a mean age of 48.0 ± 12.4 years, and 614 of them (75.0%) were women. Of the 819 patients, 786 patients underwent biopsy of one nodule, whereas 33 underwent biopsy of two nodules. Among the 852 thyroid nodules, 471 (55.3%) had echogenic foci on US, and 381 (44.7%) did not. Of the nodules with echogenic foci, punctate echogenic foci were observed in 369 (78.3%) nodules, and macrocalcifications and peripheral calcifications were present in 36 (7.6%) and 20 (4.2%) nodules, respectively. In addition, nodules with multiple different types of echogenic foci constituted 46 nodules, among which there were 41 (89.1%) nodules with punctate echogenic foci and macrocalcification, three (6.5%) with punctate echogenic foci and peripheral calcification, one (2.2%) with macrocalcification and peripheral calcification, and one (2.2%) with three types of echogenic foci. **Table 1** summarizes the US features and CNB pathology results for all nodules with echogenic foci.

**Table 1:** US features and CNB results for thyroid nodules with different echogenic focus patterns.

	Punctate echogenic foci n (%)	Macrocalcification n (%)	Peripheral calcification n (%)	Multiple different calcification types n (%)	*p*
**Gender**					
Men	78 (21.1%)	13 (36.1%)	5 (25.0%)	17 (37.0%)	0.047
Women	281 (76.2%)	23 (63.9%)	15 (75.0%)	29 (63.0%)	
**Age** (years)	47.1 ± 13.8	53.5 ± 12.1	53.3 ± 12.4	51.8 ± 14.4	0.005
**US features**					
**Diamete**r^*^(cm)	1.20 (0.90-1.70)	1.70 (1.00-2.90)	1.10 (0.80-1.95)	1.70 (1.00-3.00)	0.000
**Shape**					
AP/T >1	129 (35.0%)	9 (25.0%)	2 (10.0%)	13 (28.3%)	0.074
AP/T <1	240 (65.0%)	27 (75.0%)	18 (90.0%)	33 (71.7%)	
**Echogenicity**					
Very hypoechoic	11 (3.0%)	1 (2.8%)	0	0	0.002
Hypoechoic	331 (89.7%)	24 (66.7%)	10 (50.0%)	40 (87.0%)	
Iso/hyperechoic	21 (5.7%)	8 (22.2%)	4 (20.0%)	4 (8.7%)	
Cannot be determined^†^	6 (1.6%)	3 (8.3%)	6 (30.0%)	2 (4.3%)	
**Composition**					
Mix cystic and solid	13 (3.5%)	4 (11.1%)	0	5 (10.9%)	0.021
Solid	350 (94.9%)	29 (80.6%)	14 (70.0%)	39 (84.8%)	
Cannot be determined^†^	6 (1.6%)	3 (8.3%)	6 (30.0%)	2 (4.3%)	
**Margin**					
Smooth	34 (9.2%)	6 (16.7%)	9 (45.0%)	5 (10.9%)	0.000
Ill-defined	16 (4.3%)	0	3 (15.0%)	0	
Irregular	314 (85.1%)	30 (83.3%)	8 (40.0%)	39 (84.8%)	
ETE	5 (1.4%)	0	0	2 (4.3%)	
**CNB results**					
II	105 (28.5%)	25 (69.4%)	12 (60.0%)	28 (60.9%)	0.000
V	69 (18.7%)	1 (2.8%)	4 (20.0%)	4 (8.7%)	
VI	195 (52.8%)	10 (27.8%)	4 (20.0%)	14 (30.4%)	

US, ultrasonography; CNB, core needle biopsy; ETE, extrathyroidal extension; AP/T, anteroposterior dimension/transverse dimension ratio.

*The diameters in parentheses were the interquartile range.

^†^Echogenicity or composition cannot be determined because of echogenic foci.

Among the 471 nodules with echogenic foci, 170 (36.1%) were benign and 301 (63.9%) were malignant or suspicious for malignancy. Among the 381 nodules without echogenic foci, 172 (45.1%) were benign and 209 (54.9%) were malignant or suspicious for malignancy. There was a significant statistical difference (*p* = 0.007) in the prevalence of malignant or suspicious for malignancy lesions between nodules with and without echogenic foci. There was no significant statistical difference in the malignant or suspicious for malignancy rate between nodules with peripheral calcification and those with macrocalcification [40.0% (8/20) *vs*. 30.6% (11/36), respectively; *p* = 0.474]. The incidence of malignancy or suspicious for malignancy for nodules with different patterns of echogenic foci and the results of a comparison of these nodules with nodules with punctate echogenic foci are listed in **Table 2**. Compared with nodules with punctate echogenic foci alone, the incidence of malignancy or suspicious for malignancy for nodules with peripheral calcification, macrocalcification, or multiple different types of echogenic foci was significantly lower, with OR values of 0.265 (95% CI: 0.105–0.667; *p* = 0.005), 0.175 (95% CI: 0.083–0.368; *p* = 0.000), and 0.256 (95% CI: 0.136–0.482; *p* = 0.000), respectively.

**Table 2:** Echogenic focus patterns and incidence of malignancy or suspicious for malignancy in nodules with echogenic foci.

	II	V-VI	*p* ^*^
Type 1 (n=369)	105 (28.5%)	264 (71.5%)	0.000
Type 2^†^ (n=36)	25 (69.4%)	11 (30.6%)	
Type 3^‡^ (n=20)	12 (60.0%)	8 (40.0%)	
Type 4^§^ (n=3)	1 (33.3%)	2 (66.7%)	
Type 5^||^ (n=41)	26 (63.4%)	15 (36.6%)	

Type 1, punctate echogenic foci; Type 2, macrocalcification; Type 3, peripheral calcification; Type 4, both punctate echogenic foci and peripheral calcification; Type 5, both punctate echogenic foci and macrocalcification.

^*^p value indicates that echogenic focus patterns and the incidence of malignancy or suspicious for malignancy in nodules are associated.

^†,‡,§,||^p values indicate that different echogenic focus patterns and the incidence of malignancy or suspicious for malignancy in nodules are associated compared with punctate echogenic foci alone (p = 0.000, p = 0.005, p = 0.852, p = 0.000, respectively).

According to the ACR TI-RADS, we found no statistical difference in a recommendation for fine-needle aspiration when peripheral calcification was assigned 1 point *vs*. 2 points [40.0% (8/20) *vs*. 45.0% (9/20), respectively; *p* = 0.749].

## Discussion

There are several different patterns of thyroid echogenic foci, namely, punctate echogenic foci, peripheral calcification, macrocalcification, and multiple different types of echogenic foci. Our study showed that 55.3% of thyroid nodules exhibited echogenic foci, of which 63.9% were malignant or suspicious for malignancy. This result is consistent with the calcification rate and malignant rate in previous studies (14, 15). Ha et al. (16) found that the incidences of punctate echogenic foci, macrocalcification, and peripheral calcification in thyroid nodules with echogenic foci were 53.1%, 31.0%, and 5.4%, respectively. Wang et al. (17) reported that in nodules with calcification, 33.69% (532/1579) had microcalcification, 60.16% (950/1579) had macrocalcification, and 6.14% (97/1579) had rim calcification. Compared with our results, the presence of punctate echogenic foci was low in the cited previous studies. Additionally, the presence of macrocalcification and peripheral calcification in the cited studies was higher than that in our study. In our hospital, the indications for thyroid CNB are a diameter larger than 5 mm and nodules with more than one suspicious US feature (hypoechoic or very hypoechoic, anteroposterior dimension/transverse dimension ratio >1, ill-defined margin, irregular margin, echogenic foci). In highly anxious patients, we perform CNB if a thyroid nodule is 3–5 mm in diameter and is accompanied by at least two suspicious US features. However, this indication may have caused selection bias and resulted in differences in the incidences of the various types of echogenic foci between our and previous studies.

In the ACR TI-RADS, punctate echogenic foci are assigned 3 points, peripheral calcifications are assigned 2 points, and macrocalcifications are assigned 1 point. The presence of microcalcifications is highly suggestive of malignancy, but the association between peripheral calcification or macrocalcification and thyroid carcinoma is still controversial (18–23). Malhi et al. (9) reported that the risk of malignancy was 27% in nodules with peripheral calcification. However, the characteristics of the peripheral calcifications, such as focal interruption of rim calcifications and the presence of soft tissue outside the calcification, had no significant value when evaluating the risk of malignancy owing to high interobserver variability. Yoon et al. (24) classified peripheral calcification as stippled calcification, smooth curvilinear calcification, and irregular curvilinear calcification and categorized the lesions as arc or rim. The authors also found that the type of peripheral calcification provided no useful information for predicting malignancy. A study by Park et al. (25) of 649 nodules with macrocalcifications showed that 27.6% of the nodules were malignant. Several investigations evaluated both macrocalcifications and peripheral calcifications together. Arpaci et al. (26) retrospectively evaluated 907 nodules and suggested that eggshell calcification and parenchymal macrocalcification were associated with a higher suspicious for malignancy rate, and parenchymal macrocalcification was associated with a higher rate of malignancy compared with nodules without macrocalcification. The total rate of malignancy and the rate of suspicious for malignancy for eggshell calcification and parenchymal macrocalcification were 9.4% and 9.0%, respectively. Among 43 patients who underwent surgery, the malignant rate of nodules with eggshell calcification was 50.0% (2/4) and the malignant rate of parenchymal macrocalcification was 59.1% (13/22). Taki et al. (4) found that 43% (6/14) of nodules with peripheral calcification and 52% (15/29) of nodules with intranodular coarse calcification were diagnosed as cancer, which was confirmed surgically. Our results suggested that there was no significant statistical difference in the malignant or suspicious for malignancy rate between nodules with peripheral calcification and those with macrocalcification. However, when assigning peripheral calcifications a score of 1 point, there was no statistical difference in the rate of nodules meeting criteria for fine-needle aspiration compared with assigning peripheral calcifications a score of 2 points.

Some studies have reported an association between nodules with multiple different echogenic focus types and thyroid cancer. A multi-institutional analysis conducted by Middleton et al. (27) suggested that the risk of malignancy associated with macrocalcification, peripheral calcification, and punctate echogenic foci in solid nodules was 11.8%, 20.2%, and 35.0%, respectively. The authors also reported that the rate of malignancy for nodules with both punctate echogenic foci and macrocalcification and both punctate echogenic foci and peripheral calcification was 34.1% and 50.0%, respectively. Ha et al. (16) found that malignant rates with peripheral calcification and macrocalcification were 33.3% (7/21) and 52.9% (64/121), respectively. The authors also proved that nodules with more than one type of echogenic foci increased the risk of malignancy to 73.2% (30/41). In our study, 37.6% of the nodules with echogenic foci measured 0.5–1.0 cm in diameter, the incidence of macrocalcification and peripheral calcification in these nodules was lower than for nodules with a diameter >1.0 cm, and 83.6% had punctate echogenic foci alone. This may explain why our results differed from those in previous studies. According to our results, when evaluating nodules with multiple different types of echogenic foci, regression analysis showed that the prevalence of malignancy or suspicious for malignancy for nodules with multiple different types of echogenic foci was significantly lower than that for single punctate echogenic foci, with an OR of 0.256 (95% CI: 0.136–0.482). While peripheral calcification or macrocalcification combined with punctate echogenic foci showed a higher risk of malignancy or suspicious for malignancy than that for peripheral calcification or macrocalcification alone, the prevalence was still lower than that for punctate echogenic foci alone. Therefore, our study showed that summing the scores of various types of echogenic foci when determining the TI-RADS level may be inappropriate.

A limitation of our study was the single-institution design. The number of cases with peripheral calcification, including peripheral calcification alone and other patterns of echogenic foci accompanied by peripheral calcification, was relatively small, and large-sample data are needed to confirm our results and explore the different risks of malignancy in mixed solid and cystic nodules and solid nodules. Second, our approach to punctate echogenic foci and peripheral calcifications differ slightly from the ACR approach (12, 28), and this difference may have led to bias in the results. Third, our work did not suggest specific modifications to ACR TI-RADS, and we did not test the recommendations against a modified version of ACR TI-RADS; we expect to explore this problem in future work. Furthermore, our results were limited by the fact that lesion diagnosis was according to CNB, which may lead to false-negative or false-positive results. Finally, the cases in our study were from some years ago, and recent technological advances may lead to different results in future similar studies.

## Conclusion

We found no significant statistical difference for the risk of malignancy or suspicious for malignancy rate between peripheral calcification and macrocalcification in thyroid nodules. We observed that nodules with multiple different types of echogenic foci were not associated with higher malignant or suspicious for malignancy rates compared with nodules with punctate echogenic foci alone. Thus, in the ACR TI-RADS, assigning peripheral calcifications 2 points and macrocalcifications 1 point and summing the scores of the various detected types of echogenic foci may need further verification by multiple centers and a large-sample study.

## Data availability statement

The raw data supporting the conclusions of this article will be made available by the authors, without undue reservation.

## Ethics statement

This study was reviewed and approved by Peking University First Hospital National Unit of Clinical trials Ethics Committee. The patients/participants provided their written informed consent to participate in this study.

## Author contributions

RL wrote the manuscript. LC designed the study. RL, ZL and LC were involved in the data collection. RL, ZL and XW analyzed the data. All authors contributed to the article and approved the submitted version.

## Funding

This work was supported by the Research Fund of Peking University First Hospital (grant2017CR05).

## Conflict of interest

The authors declare that the research was conducted in the absence of any commercial or financial relationships that could be construed as a potential conflict of interest.

## Publisher’s note

All claims expressed in this article are solely those of the authors and do not necessarily represent those of their affiliated organizations, or those of the publisher, the editors and the reviewers. Any product that may be evaluated in this article, or claim that may be made by its manufacturer, is not guaranteed or endorsed by the publisher.
